# Clinical added value of triplicate electrocardiograms: a review of 505 triplicate sets

**DOI:** 10.1186/s40959-026-00472-4

**Published:** 2026-03-18

**Authors:** Michael S. Ewer, Hyeon-Ju Ryo Ali, Nicolas L. Palaskas, Jay Herson, Anita Deswal

**Affiliations:** 1 Department of Cardiology, Division of Internal Medicine, The University of Texas, MD Anderson Cancer Center, Houston, 77030 Texas USA; 2 The Department of Biostatistics, Johns Hopkins Bloomberg School of Public Health, Baltimore, MD USA

## Abstract

Triplicate electrocardiograms (ECGs) are routinely included in clinical trials where concern of agent-induced electrophysiological alterations arise. Three electrocardiograms (triplicate ECGs) are obtained over a time interval of less than 10 min with the intent to increase recognition of changes, and to provide increased data for measurement and review of physiologic intervals. We undertook a prospective review of over 500 sets of triplicate ECGs to evaluate the added value of two additional tracings over a single ECG. Our review was done exclusively to evaluate the actual ECG tracings and not the reproducibility of the electronic interpretive algorithms. All ECGs were reviewed by one of the cardiologists over a four-month period. We found no meaningful changes in R-R, P-R, or Q-T intervals, nor any change in the QRS duration that was observed during the *≤* 10-minute period of data acquisition; rare supraventricular and ventricular ectopy was noted in 4 of the 505 triplicate sets. The routine inclusion of a requirement for triplicate ECGs should be revaluated to assess the added value to the patient or clinical research as well as potential burdens to the patient and financial impact.

## Introduction

The electrocardiogram (ECG), reflecting the summation of electrical events that accompany action potentials and repolarization changes as recorded on the surface of the body dates back more than 120 years. Einthoven defined the various segments of the surface electrocardiogram in 1903 and was later awarded a Nobel Prize for this work [1]. The value of the surface ECG remains relevant, perhaps in part due to the limitations of more modern cardiac imaging techniques in defining some electrical phenomenon and intervals; more modern techniques are better suited to evaluate cardiac function. The ECG continues to be the method of choice for evaluating cardiac rhythm as well as the timing and duration of cardiac intervals that continue to play a large role in diagnosing and predicting cardiac events [2]. The ECG and its variants have gone beyond use by clinicians to evaluate or quantitate pharmaceutical-related alterations in electrocardiographic parameters as an important component of drug development.

Additionally, applications have evolved that allow individuals or their caregivers to record and transfer the surface electrical pattern, thereby allowing remote screening of cardiac rhythm disturbances [3]. The modern electrocardiogram is inexpensive, rapidly performed, and generally read with the help of dedicated interpretative algorithms. In addition to the timing of intervals and the evaluation of cardiac rhythms, it provides information that suggests metabolic or structural changes seen with ischemia, infarction, cardiac toxicity of cancer therapies, inflammation, infiltration, or even variations in shifting cardiac position. Alterations in the ECG with exercise remain a crucial diagnostic tool for the evaluation of ischemia-related metabolic changes. Because of the predictive value of the 12-lead ECG they are now part of screening for interventions that go beyond the cardio-pulmonary system. They form part of preoperative evaluation for most surgeries requiring general anesthesia, they form part of routine physical examinations, and they play an increasing role in evaluating cancer patients before, during and after interventions for disease control or cure. Concern regarding prolongation of the corrected QT interval (QTc) has garnered a special concern, as the QTc prolongation may be a prequel to drug-induced rhythm disturbances leading to ventricular tachycardia of the *torsades de pointes*, a condition that can be fatal [4]. Clinical investigations of new agents, where the corrected QT interval may be prolonged, now incorporate extensive monitoring. A typical protocol might also require additional triplicate sets at 15-minute intervals, both pre-treatment and sometimes during the administration of the investigational agent. The International Council for Harmonization of Technical Requirements for Pharmaceuticals for Human Use (ICH) E14 guideline, published in May 2005, mandates a “thorough QT/QTc” study powered to identify drug-induced QTc prolongation greater than ≥ 5 milliseconds or an upper bound of 95% confidence interval ≥ 10 milliseconds). This was implemented by the Food and Drug Administration for new drugs with systemic bioavailability and now typical research protocol includes pre-treatment and post-treatment triplicate ECGs with limited data to support this practice https://www.fda.gov/media/71372/download.

Such monitoring is undertaken to seek and quantitate changes that may ensue as a direct or indirect result of the administration of a new agent where the full spectrum of effects and events has not yet been determined. This is especially relevant in cancer patients who may demonstrate baseline prolongation of the QTc because of their underlying conditions or concomitant use of agents known to prolong the QTc [5]. Many of these protocols require triplicate ECGs, defined as three separate tracings recorded less than five minutes apart, done on a single patient. This study was undertaken to explore the added value to the patient of triplicate ECGs compared to single ECGs, and to evaluate the overall benefit of such monitoring in a tertiary cancer center.

## Materials

We performed a prospective observational cohort study at MD Anderson Cancer Center between July and October 2025. Patients with triplicate ECGs, defined as 3 ECGs performed within a 10-minute interval, were included in the study. In most instances these were done as the requirements of oncologic protocols. Pediatric ECGs, defined as studies on individuals aged less than 18 years of age at the time of ECG were excluded. While the tracings underwent initial electronic interpretation, it was not our goal to scrutinize or evaluate the consistency, precision or accuracy of the electronic reading or the accompanying algorithm-provided measurements of cardiac intervals as presented on the preliminary recordings. We felt that there likely would be variability between the available interpretative systems and perhaps even among the individual components of any of the available systems. We were interested only in how three ECGs recorded within the defined time frame might add useful patient-related information that could have relevance for either the clinical care of patients or for research purposes. Thus, the goal was to evaluate biologic change in a patient parameter that would have the potential to addvalue for for research or patient care purposes.

One of the three clinicians who are actively involved in ECG overreading, modifying the readings as appropriate, and finalizing the reports reviewed the ECGs on a single day for this study. The clinicians agreed to scrutinize the individual ECGs in those instances where triplicate ECGs were ordered on the days of their assigned reading and to evaluate each set for meaningful variants among the three tracings. A meaningful variant or alteration might include rhythm alterations, conduction alterations, or repolarization changes; these changes were to be recorded on the final report of the particular ECG in question. In many instances multiple sets of three ECGs were ordered and interpreted on a single day and that information was also recorded. When present, each multiple set of three ECGs in a single day was treated separately, as ascertaining changes over longer periods of time was not our goal. Our criteria for identifying variance among a set of three ECGs was any meaningful alteration in rhythm, conduction pattern, or repolarization phenomena. These included timing of intervals (PR interval, QRS duration, and QT interval and cardiac rate). The QT interval variation before correction for cardiac rate was assessed. When variance among the three determinations was suggested by the electronic interpretation, manual measurement with correction and confirmation was undertaken. We looked for observable changes in these parameters that would reflect biologic or pharmacologic variance in rhythm, conduction, repolarization and the timing of intervals that would be identified as a change. Each of the three readers over-read the ECGs on his or her assigned date; while the findings were shared among the group, each set of ECGs was reviewed only by one physician.

The computer-generated measurements were reviewed by the readers, but only variation in these measurements that could be appreciated by the readers and deemed clinically meaningful were to be noted as a difference. Outlying computer-generated discrepances that were not confirmed by manual over-reading by the cardiologists were deemed to be the result of weakness in the algorithms or measurement variation and were not considered a meaningful change. In instances where several sets of three ECGs were recorded on a single day, changes from one set to another were neither noted nor recorded; only changes among the three ECGs in any triplicate set comprise the findings of this study.

As we had little preliminary information to estimate the sample size, we chose as a preliminary sample size of 500 sets of three, with the opportunity to expand the number after our initial review. In order to test the null n hypothesis of a discordant rate of 1% vs. an alternative hypothesis of 5% a sample size of 500 yields a power of 0.999 in a one-sided hypothesis test performed at significance level 0.025. The triplicate ECG review protocol was reviewed by the internal institutional review board. Our final sample consisted of 505 sets of triplicate ECGs, or a total of 1515 individual tracings.

## Results

The 505 sets of three ECGs were recorded during an interval of less than 10-minutes were obtained over the 4-month period for a total of 1515 individual tracings used in this analysis. During that time 6359 individual ECGs were reviewed by the three clinicians (ME, NLP, HJRA). Approximately 24% of the total ECGs performed and interpreted by the study clinicians during this timeframe were part of the triplicate ECG evaluation, drawing attention to the number of triplicate studies performed in a large research facility.

Of our series of 505 sets of triplicate ECGs, 239 sets comprised a single set of triplicates on the day they were recorded. The remainder of the studies involved several determinations ranging from 2 to 8 triplicate sets done in a single day; there were 95 sets of 2 or more triplicate determinations (Table 1).

**Table 1:** Breakdown of the number of triplicate ECGs on any day

Number of triplicate studies in any patient on a single day	1	2	3	4	5	6	7	8	
	239	64	11	7	5	5	2	1	
Makeup of the triplicate sets	239	128	33	28	25	30	14	8	505

In our population of 505 triplicate sets there were 4 instances (0.8%) where a change among the three ECGs was noted. Rhythm disturbance was the change noted in all 4 instances: one had two ventricular premature ventricular complexes in one of the three tracings but none in the other two; one set showed a single episode of coupled atrial premature complexes in two of the three tracings with none in the third, and in the remaining two, two of the three tracings showed 2 premature atrial complexes while the third in each did not show ectopy. The dysrhythmias found are summarized in Table 2. No meaningful variation in the scrutinized intervals within the triplicate cardiograms, unrelated to the noted dysrhythmia, was observed. QT intervals were not recorded separately for the purposes of this study beyond what was provided by the computer interpretation but were observed as part of the over-reading process. No QT changes were appreciated within the timeframe of the triplicate recording in any of the 503 sets reviewed. Within the limits of how this study was performed the QT remained constant over the limited time-period. Small variances in the computer-generated and reported values of intervals were seen and deemed not to reflect meaningful physiologic or pharmacologic change. The clinicians did not feel any of these finding reflected a material alteration in either the baseline electrocardiogram or was related to the reason for performing the triplicate studies.

**Table 2:** Annotated findings

1. One ECG of three tracings showed two isolated PVCs; the other two had no ectopy.
2. One episode of a couplet of atrial premature complexes in each of 2 of the three tracings; the third did not show ectopy.
3. One of a set of three had two PACs, the other two had none.
4. One of a set of three had two PACs, the other two had none.

## Discussion

We recognize several possibilities as to why triplicate ECGs might have relevance: the first being that real clinically material changes might be taking place. In considering the QT interval, antiarrhythmic drugs, the list of causes of QT prolongation includes medications of many classes including antibiotics, cholesterol-lowering agents, antidepressants and diuretic medications; in the cancer population anticancer treatment and supportive may be implicated [6]. As the interval between the three ECGs was less than 10 min, no QT-prolonging medications were administered. Our study did not show QT-interval changes that occurred within the timeframe of the recording; no clinically meaningful findings in any of the parameters were noted, other than the described incidental rhythm disturbances.

The second possibility is that despite non-meaningful real changes, triplicate ECGs could allow for obtaining a mean value for computer-assisted measurements including the R-R interval, the P-R interval, the Q-T interval, and the QRS duration that might have a meaningful research component. We find this justification for triplicate ECGs troubling in that no substantial difference, based on our review of 505 triplicate sets was found in our review of the individual tracings. In instances where deviations were initially reported on the computer-generated report, all were the result of artifacts or errors introduced by the electronic interpretation; none identified real change. Had the mean value been taken of the three readings, where one was an erroneous outlier, the reported value would have depicted an incorrect value that could have had clinical impact or have potentially triggered a false-positive event in a subject who did not experience a change. Hopefully electronic ECG evaluation will evolve with improved algorithms, perhaps with the incorporation artificial intelligence, thereby mitigating errors introduced through the inclusion of outliers.

Only one other trial has evaluated the role of replicate ECGs for improving detection of drug-induced QTc prolongation to our knowledge. This was a retrospective review of QT intervals obtained from a randomized controlled study evaluating the effect of moxifloxacin, including 17,838 ECGs from 124 subjects. Six replicate ECGs were recorded within 10 min pre- and post-treatment. The within-subject standard deviation for QTcF was 9.5 milliseconds with 1 replicate and 7.3 milliseconds with 3 replicates which is a small clinical difference. This finding is consistent with our study that with our total of 505 triplicate ECGs, there were no meaningful changes detected [7].  

Additional concerns are related to the cost of triplicate ECG and patient inconvenience. The automatic triggering of a requirement for triplicate ECGs, either for research purposes or clinical considerations, should be reconsidered and justified when there is clinical concern for real physiologic, pharmacologic, or metabolic change occurring over a short period of time. As an alternate, a longer single tracing in those instances where changes are of concern might provide a better representative value over obtaining a mean of three 6 s individual ECG recordings.

## Conclusion

In our series of 505 sets of triplicate ECGs no added value for our patients could be found because of the second and third tracing of a triplicate set performed over a short time interval. Within the limits of the standard 12-lead ECG, no differences in the measured intervals, conduction, or repolarization were noted. No data that could affect the adjudication of the research data or adverse events reporting in a trial was observed. Additionally, obtaining mean values of imperfect interval data may introduce errors that could result in confusion and incorrect adjudication of events. The routine inclusion of triplicate ECG should be reevaluated to ensure meaningful added value while minimizing inclusion of sub-optimal or false positive data that could be adjudicated as a meaningful change.

## Data Availability

All data supporting the findings of this study are available within the paper.
